# Assessing the impact of migraine on benign paroxysmal positional vertigo symptoms and recovery

**DOI:** 10.1186/s12883-024-03606-2

**Published:** 2024-05-02

**Authors:** Seda Çakır, Aysenur Sahin, Ozlem Gedik-Soyuyuce, Zeynep Gence Gumus, İbrahim Sertdemir, Nazım Korkut, Pınar Yalınay Dikmen

**Affiliations:** 1 Department of Neurology, Acıbadem University School of Medicine, İçerenköy. Kayışdağı Cad. No: 32. Ataşehir, İstanbul, 34752 Turkey; 2 Department of Audiology, Acıbadem Maslak Hospital, İstanbul, Turkey; 3 Department of Biostatistics and Bioinformatics, Acıbadem University, İstanbul, Turkey; 4 Department of Otorhinolaryngology, Acıbadem Maslak Hospital, İstanbul, Turkey

**Keywords:** Benign paroxysmal positional vertigo, BPPV, Vertigo, Dizziness, Migraine

## Abstract

**Background:**

During episodes of benign paroxysmal positional vertigo (BPPV), individuals with migraine, compared with individuals without migraine, may experience more severe vestibular symptoms because of their hyperexcitable brain structures, more adverse effects on quality of life, and worse recovery processes from BPPV.

**Methods:**

All patients with BPPV were assigned to the migraine group (MG, *n* = 64) and without migraine group (BPPV w/o MG, *n* = 64) and completed the Vertigo Symptom Scale (VSS), Vertigo Dizziness Imbalance Symptom Scale (VDI-SS), VDI Health-Related Quality of Life Scale (VDI-HRQoLS), Beck Anxiety Inventory (BAI), and Beck Depression Inventory (BDI) at the time of BPPV diagnosis (baseline) and on the one-month follow-up. Headache Impact Test-6 and Migraine Disability Assessment Scale were used for an assessment of headache. Motion sickness was evaluated based on the statement of each patient as present or absent.

**Results:**

Compared with the BPPV w/o MG, the MG had higher VSS scores at baseline [19.5 (10.7) vs. 11.3 (8.5); *p* < 0.001] and on one-month follow-up [10.9 (9.3) vs. 2.2 (2.7), p < 0.001]; experienced more severe dizziness and imbalance symptoms based on the VDI-SS at baseline (61.9% vs. 77.3%; *p* < 0.001) and after one month (78.9% vs. 93.7%, *p* < 0.001); and more significantly impaired quality of life according to the VDI-HRQoLS at baseline (77.4% vs. 91.8%, *p* < 0.001) and after one month (86.3% vs. 97.6%, *p* < 0.001).

On the one-month follow-up, the subgroups of patients with moderate and severe scores of the BAI were higher in the MG (39.2%, *n* = 24) than in the BPPV w/o MG (21.8%, *n* = 14) and the number of patients who had normal scores of the BDI was lower in the MG than in the BPPV w/o MG (67.1% vs. 87.5%, *p* = 0.038).

**Conclusion:**

Clinicians are advised to inquire about migraine when evaluating patients with BPPV because it may lead to more intricate and severe clinical presentation. Further studies will be elaborated the genuine nature of the causal relationship between migraine and BPPV.

**Supplementary Information:**

The online version contains supplementary material available at 10.1186/s12883-024-03606-2.

## BAckground

Migraine is a vastly prevalent and disabling neurological disorder worldwide [1]. It is not defined as merely headache, which is only one of the phases of a migraine attack. In addition to pain, the several symptoms during migraine attacks reflect a complex pathophysiology and the diffuse involvement of multiple neural networks and anatomical regions, such as the autonomic, affective, cognitive, and sensory systems, as well as the brainstem [2]. Compared with individuals without migraine, those who have migraine have brains that are hyperexcitable from the influence of genetic and epigenetic factors and exhibit distinct characteristics in their ability to cope with internal and external stimuli that disrupt homeostasis [3]. Functional imaging and neurophysiological studies have provided concrete proof that the brains of individuals with migraine exhibited increased responsiveness to sensory stimuli, even during the interictal phase [3, 4]. Moreover, compared with individuals without migraine, those with migraine were reported to exhibit increased activation in the primary visual cortex and the other visual processing regions, such as the lateral geniculate nucleus and the motion-responsive middle temporal cortex, when exposed to visual stimuli [5, 6]. The clinical implication of the results of functional imaging and electrophysiological studies was that individuals experienced migraine attacks when exposed to internal (e.g., menstruation, sleep disturbances, skipping meals, and stress) or external (e.g., changes in air pressure, crowded environments, entering poorly ventilated spaces, and tying up hair) triggers that surpass their allostatic loads [7]. In this context, the ability of individuals with migraine to cope with stressors was assumed to differ from that of individuals without migraine [2, 8–10]. The exact mechanisms of the increased cortical responsiveness in migraine (i.e., increased excitability or decreased inhibition in the brain and central or peripheral origin) have not been fully elucidated and remain a topic of debate [11].

Benign paroxysmal positional vertigo (BPPV) is the most common cause of vertigo [12]. Individuals with migraine were reported to be more likely to experience BPPV [13–16]. Owing to the maladaptive or hypersensitive brains in individuals with migraine, symptoms that may arise from stimulation or dysfunction of the peripheral vestibular system can be severe and bothersome [8–10]. In this context, BPPV could be an acute trigger for the disruption of the peripheral vestibular system balance in individuals with migraine. Compared with individuals without migraine, those with migraine may experience more severe vestibular symptoms during a BPPV attack because of their hyperexcitable brain structures, experience a greater impact on their quality of life (QoL), and have a longer recovery process.

In this study, we hypothesized a heightened severity of vestibular symptoms, such as vertigo and dizziness, during episodes of BPPV among individuals with migraine and vestibular migraine (VM), which had been increasingly recognized in recent years as the most common cause of spontaneous episodic vertigo and is the second most common vestibular disorder following BPPV. Furthermore, we anticipated a more negative impact on overall QoL and a less effective recovery from BPPV in this group than in individuals without a history of migraine. To test our hypothesis, we aimed to compare the vertigo, dizziness, and QoL scales at the time of BPPV diagnosis and on one-month follow-up between individuals with migraine and those without migraine. In addition, we aimed to test the validity of our hypothesis in the subgroup of patients with VM.

## Methods

### Study population and study design

In this study, 128 consecutive patients who had BPPV that presented as dizziness or vertigo and were clinically evaluated at the ear nose throat (ENT) and neurology outpatient clinics of Acıbadem Maslak Hospital between April 2022 and November 2022 were prospectively recruited for six months. The diagnosis of BPPV was confirmed based on the results of videonistagmography (VNG), which was done at the audiology laboratory. Clinical assessment included complete medical history and neurootological, neurological, and physical examinations. The study was conducted in accordance with the ethical principles stated in the Declaration of Helsinki. Institutional review board approval was granted by Acibadem University School of Medicine (2022–02/17). Written informed consent was obtained from all the participants prior to enrolment.

**Fig. 1:**
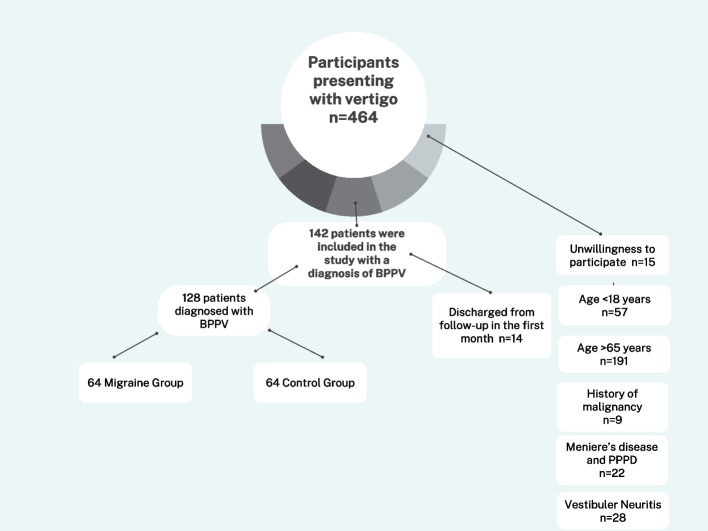
Flow diagram

The inclusion criteria were age ≥ 18 years and < 65 years, literate, provision of consent, and at least one year of headache in patients who had migraine. The diagnosis of migraine was made by a neurology specialist according to the International Classification of Headache Disorders, third edition [17]. The exclusion criteria were the presence of conditions that can affect cognitive performance; severe physical illness or clinical laboratory findings that indicated a serious illness, such as malignancy; history of severe neurological disease, such as cerebrovascular disease; being under the influence of psychoactive substances; history of debilitating central or peripheral vestibular diseases, such as vestibular neuritis, persistent postural perceptual dizziness (PPPD) and having Meniere’s disease. During the one-month follow-up period, the participants did not receive any new treatment to suppress vestibular symptoms or migraine. Based on the aforementioned criteria, patients were excluded because of unwillingness to participate (*n* = 15), age < 18 years (*n* = 57), age > 65 years (*n* = 191), history of malignancy (*n* = 9), the presence of Meniere’s disease and PPPD (*n* = 22) and vestibular neuritis (*n* = 28), being lost to follow-up within one month (*n* = 14). Therefore, a total of 128 eligible patients who met the criteria were included in the study (Fig. 1). Among the patients with BPPV who were included in the study, those with migraine were classified as the migraine group (MG) and those without migraine were classified as the BPPV without migraine group (BBPV w/o MG).

### Assessments

After being diagnosed by the participating experts in the study, all patients filled out the standardized questionnaires and measurement tools during the interview with the same neurology research assistant (SC). The patients were interviewed twice; the first was during the diagnosis of BPPV (baseline) and lasted for approximately 60 min, and the second was during the follow-up visit after one month and lasted for about 30 min. During the first interview, the demographic characteristics of all patients were recorded. The patients completed the data form on the clinical features of BPPV and migraine, as well as the other clinical scales that were used in the study (Additional file 1). Individuals experiencing 4–14 headache days per month were categorized as having frequent episodic migraine (EM), while those with 0–3 headache days per month or fewer were classified as having infrequent episodic migraine (EM). Patients experiencing ≥ 15 headache days per month were classified as chronic migraine (CM).

Risk factors of BPPV were also questioned in the data form. Recent trauma was defined to injuries to the head and neck regions within the previous month. The presence of viral or bacterial infections affecting the upper or lower respiratory system within the past month was investigated as a potential risk factor. The term "prolonged rest" was employed to characterize bed rest following surgery within the preceding month. Heavy alcohol consumption was defined to the excessive intake of alcohol within the 3-day period before the appearance of BPPV-related complaints, typically exceeding recommended daily limits or surpassing moderate drinking guidelines. Vitamin deficiency was characterized by a blood level of 25-hydroxy vitamin D below 25 ng/mL. Stress positivity was evaluated as the patient expressing his or her stress level as normal or increased following the negative life events experienced in the last month. Acute insomnia was characterized by disruptions in sleep continuity, involving difficulty in initiating and/or maintaining sleep, and occurring for a duration of at least three days per week, lasting anywhere between one week and three months.

For the evaluation of the clinical features of vertigo, the Vertigo Symptom Scale (VSS), Vertigo Dizziness Imbalance Symptom Scale (VDI-SS), and Health-Related Quality of Life Scale (VDI-HRQoLS) were used. For the evaluation of headache, the Headache Impact Test (HIT-6) was applied. For the assessment of migraine-related disability, the Migraine Disability Assessment Scale (MIDAS) was used. The Beck Anxiety Inventory (BAI) and Beck Depression Inventory (BDI) were used to assess anxiety and depression symptoms. In the absence of a clinically validated and reliable Turkish scale for evaluating motion sickness, participants were classified as either having a history of motion sickness (present) or not having it (absent), relying on their past or current experiences.

Motion sickness was evaluated based on the statement of each patient as present or absent. The MG were given a headache diary during the baseline interview. All patients were invited for a follow-up appointment after one month for repeat completion of the clinical scales and collection of the headache diaries from the MG.

### Vertigo symptom scale

The VSS was developed for the assessment of vertigo symptoms [18, 19]. The VSS short form comprised 15 items, which were evaluated on a 0–4 Likert-type scale, based on the frequency during the past month (0: never, 1: very rarely, 2: most of the time, 3: often- every week, or 4: very often- most days). The patients were asked to choose the most appropriate response that reflected their condition. The total score ranged from 0 to 60; a score of ≥ 24 was classified as severe vertigo, and a score of < 24 was classified as mild vertigo.

The VDI comprised two subscales, as follows: the VDI-SS, which contained 14 items, and the VDI-HRQoLS, which contained 22 items. Each item was classified into five subcategories using a 0–5 Likert-type scale (0: always, 1: most of the time, 2: often, 3: sometimes, 4: very rarely, and 5: never). The patients were asked to select the most appropriate response that reflected their condition. The total score ranged from 0 to 100%; a score of 100% indicated no symptoms or no impact on the QoL, whereas a score approaching 0% indicated worsening of symptoms and QoL.

### Headache impact test

The HIT-6 evaluates the impact of headaches on an individual’s life. It comprises six subdomains, including pain, social functioning, role functioning, vitality, cognitive functioning, and psychological distress caused by pain [20, 21]. The total score ranged from 36 to 78, with higher scores indicating a greater impact. The HIT-6 score was categorized into four groups, such as ≤ 49 (little to no impact), 50–55 (partial impact), 56–59 (significant impact), and ≥ 60 (severe impact).

### Migraine disability assessment scale

The MIDAS had been used to evaluate the impact of migraine on the performance of daily activities in the past three months, based on the answers to five questions in three areas, such as work or school, household chores, and activities that are related to family, social life, and leisure [22, 23]. In this study, the scores were categorized based on the severity of the disability caused by migraine attacks, as follows: 0–5 points for no or very mild disability, 6–10 points for mild disability, 11–20 points for moderate disability, and > 21 points for severe disability.

### Beck anxiety inventory

The BAI is a self-reported inventory that assesses the frequency and severity of anxiety symptoms [24, 25]. It comprises 21 symptom categories, each with four response options. Each item is scored from 0 to 3. The maximum possible score on the scale is 63. The total score was categorized as follows: 0–7 points for no anxiety, 8–15 points for mild anxiety, 16–25 points for moderate anxiety, and 26–63 points for severe anxiety.

### Beck depression inventory

The BDI is one of the commonly used self-reported instruments in research and daily practice [26, 27] and comprises 21 questions. The patients were asked to choose the most appropriate response that reflected their current state. The total score ranged from 0 to 63. The results were categorized as follows: 0–9 indicated no or minimal depression, 10–18 indicated mild depression, 19–29 indicated moderate depression, and 30–63 indicated severe depression.

### Statistical analysis

The primary outcome variables were the changes in the VSS, VDI, and VDI-HRQoLS from baseline to follow-up at one month. In our study, two types of comparative statistics were conducted to investigate the severity of the BPPV symptoms in the MG and the impact of having migraine on BPPV recovery. First, the severity of vestibular symptoms and QoL were compared between the MG and the BPPV w/o MG. In addition, the BPPV recovery process was evaluated by comparing the three scales between baseline and one-month follow-up in each group.

Statistical analysis was carried out using RStudio software V2022.12.0 (RStudio Team, 2022) and the R programming language V4.2.2 (R Core Team, 2022) with the aid of R-based packages. All the analyses were performed on the available data. A priori statistical power calculation was conducted. The sample size was based on the available data. The normality of data was assessed by the Shapiro–Wilk test. Hypothesis testing was two-tailed. For comparisons between groups, T-test, or Mann–Whitney U-test was used for numerical variables, whereas chi-square test and two-sample proportion test were used for categorical data. Posthoc pairwise comparisons were performed using Bonferroni-corrected Mann–Whitney U-test. The relationship between numerical variables was explored using Spearman’s rank correlation coefficient. The data were expressed as number and percentage for categorical variables. Numerical variables was expressed as mean, standard deviation, range, median, and interquartile range according to parametric or nonparametric distribution properties. A value of *p* < 0.05 was considered statistically significant.

## Results

### Demographic features

Table 1 presents the demographic data of all participants. The mean age was significantly lower in the MG than in the BPPV w/o MG [39.1 years (10.2 years) vs. 44.6 years (9.5 years), *p* = 0.002]. The MG had a preponderance of women (*n* = 55, 85.9%), whereas the proportion of women in the BPPV w/o MG was lower (*n* = 36, 56.2%) (*p* < 0.001). The participants in the MG (*n* = 64) were divided into the VM (*n* = 26) and nonVM (*n* = 38) groups; there were no differences in the demographic data between the two groups.

**Table 1:** Demographic features of the participants

	**MG (*N* = 64)**	**BPPV w/o MG (*N* = 64)**	***p***	**Non-VM** **Group (*N* = 38)**	**VM Group (*N* = 26)**	***p***
Female (N, %)	55 (85.9)	36 (56.2)	< 0.001*	32 (89.4)	23 (88.4)	0.908
Age (mean, SD)	39.1 (10.2)	44.6 (9.5)	< 0.001*	40.6 (9.4)	36.7 (11.0)	0.147

*N* Number of participants, *MG* Migraine group, *BPPV w/o MG* Benign paroxysmal positional vertigo without migraine group, *VM* Vestibular migraine, *SD* Standard deviation

^*^p < 0.05

### Migraine features

#### Migraine group

In this study, 15.6% (*n* = 10) had migraine with aura (MWA) and 84.4% (*n* = 54) had migraine without aura (MWoA). In 81.3% (*n* = 52) of patients, a diagnosis of migraine was previously known and 65.6% (*n* = 42) had a family history of migraine. The mean duration of migraine was 13.5 years (10.7 years). Migraine patients were categorized into three groups based on the frequency of their headaches: infrequent EM (81.2%, *n* = 52), frequent EM (7.8%, *n* = 5) and CM (11.0%, *n* = 7). In our study, 89% of the participants reported experiencing headache frequency within the range of EM. The mean and median MIDAS scores in the MG were 14.4 (19.6) and 9 (3; 21), respectively. In the MG, the mean HIT-6 score was 57,7 ± 9,0 at baseline and 54,7 [2, 9] on the first month. At baseline, 21.8% (*n* = 14) of the MG was under a prophylactic medication for migraine. In preceding month, the mean and median of monthly headache days (MHDs) and number of days of acute attack medication intake were [5.0 (6.4) - 3 (1; 5.25)] and [4.7 (7.1) - 3 (1; 5.0)], respectively. On the first month of follow-up based on the headache diaries of the patients, the mean and median MHDs and number of days of acute attack medication intake were [5.2 (6.1) - 4 (1.75;7.25)] and [4.3 (6.3) – 3 (0.75;4.25)], respectively.

### Comparison of the vestibular migraine and nonvestibular migraine groups

In the VM group, 19.2% (*n* = 5) had MWA and 80.8% (*n* = 21) had MWoA. In the nonVM group, 13.2% (*n* = 5) had MWA and 86.8% (*n* = 33) had MWoA. A pre-existing diagnosis of migraine was identified in 92.3% (*n* = 24) of individuals in the VM group, and a family history of migraine was reported by 80.7% (*n* = 21). In contrast, in the nonVM group, 73.6% (*n* = 28) had a previously known migraine diagnosis, and 55.2% (n = 21) reported a family history of migraine. Family history of migraine was statistically different between two groups (*p* = 0.04).

The average durations of migraine in the VM and nonVM groups were 9.0 years (8.3 years) and 15.7 years (11.5 years), respectively (*p* = 0.04). EM in the VM and nonVM groups was infrequent in 73.0% (*n* = 19) and 86.7% (*n* = 33), respectively, and was frequent in 11.5% (*n* = 3) and 5.2% (*n* = 2), respectively. CM was diagnosed in 15.3% (*n* = 4) and 7.8% (*n* = 3) of the VM and nonVM groups, respectively. There were no significant differences in the occurrences of EM and CM between the two groups (*p* = 0.59).

At baseline, the mean MHDs for the preceding month did not differ between the two groups (*p* = 0.312). On one-month follow-up, the median MHD was significantly higher in the VM group than in the nonVM group [5 (3; 8) vs. 3 (1; 4), *p* = 0.02]. The median MIDAS score was significantly higher in the VM group than in the nonVM group [15 (6; 24) vs. 6 (3; 15), *p* = 0.04]. There were no significant differences in the HIT-6 scores at baseline [59.3 ± 8,3 vs. 56.8 ± 9,5; *p* = 0.270] and on one-month follow-up [56.1 ± 8.3 vs. 53.8 ± 9.9; *p* = 0.32] between the VM and nonVM groups.

### Benign paroxysmal positional vertigo features

#### Migraine group

Table 2 summarizes the BPPV-related data for the MG and BPPV w/o MG. Among the patients with BPPV, the affected ear was classified as the right, left, or bilateral. There was no significant difference in the affected ear between the two groups (*p* = 0.278). BPPV was classified as affecting the posterior, horizontal, anterior, or mixed canals. In both groups, the posterior canal (PC) was most frequently affected. The proportion of patients in whom the PC was affected was not significantly different between the groups [MG (*n* = 52, 81.2%) vs. BPPV w/o MG (*n* = 43, 67.1%); *p* = 0.085].

**Table 2:** BPPV-related data of the MG and BPPV w/o MG groups

	**MG (*N* = 64)**	**BPPV w/o MG (*N* = 64)**	***p***
**Affected Side of Ears (N, %)**			
**Right**	20 (31.25)	19 (29.7)	0.278
**Left**	24 (37.5)	17 (26.6)	
**Bilateral**	20 (31.25)	28 (43.7)	
**Affected Canal (N, %)**			
**Posterior**	52 (81.25)	43 (67.1)	0,085
**Horizontal**	6 (9.3)	17 (26.5)	
**Anterior**	1 (1.5)	1 (1.5)	
**Mixed**	5 (7.8)	3 (4.6)	
**History of BPPV (N, %)**	47 (73.4)	45 (70.3)	0.844
**Number of Repositioning Maneuvers (Median, IQR)**	2 (1; 3)	2 (1; 2)	0.250
**Risk Factors (N, %)**			
**Recent trauma**	10 (15.6)	1 (1.5)	0.011*
**Recent fall**	4 (6.25)	2 (3.1)	0.675
**Recent infection**	9 (14.0)	9 (14.0)	1.0
**Motion sickness**	53 (82.9)	35 (54.7)	< 0.001*
**Prolonged rest**	0	2 (3.1)	0.476
**Exercise**	4 (6.25)	11 (17.1)	0.099
**Heavy alcohol consumption**	0	5 (7.8)	0.068
**Vitamin D deficiency**	19 (29.6)	18 (28.1)	1.0
**Stress**	8 (12.5)	16 (25)	0.112
**Acute insomnia**	2 (3.1)	3 (4.6)	1.0

*N* Number of participants, *BPPV* Benign paroxysmal positional vertigo, *MG* Migraine group, *BPPV w/o MG* Benign paroxysmal positional vertigo without migraine group, IQR: Interquartile range as Q1:Q3

^*^p < 0.05

In our cohort, most participants had a previous history of BPPV [MG (*n* = 47, 73.4%) vs. BPPV w/o MG (*n* = 45, 70.3%); *p* = 0.844]. The median number of repositioning maneuvers was similar between the two groups [MG 2 (1; 3) vs. BPPV w/o MG 2 (1; 2), *p* = 0.25]. Regarding the BPPV risk factors, recent history of trauma (15.6% vs. 1.5%, *p* = 0.01) and past or current history of motion sickness (84.3% vs. 53.1%, *p* < 0.001) were significantly more prevalent in the MG than in the BPPV w/o MG.

### Description of vertigo or dizziness

Table 3 shows the answers of the participants, according to the Barany Society classification of vestibular symptoms in the International Classification of Vestibular Diseases [28]. Visual stimulus-triggered vertigo (48.4% vs. 26.5%, *p* = 0.02) and dizziness triggered by head movement (82.8% vs. 65.6%, *p* = 0.04) were significantly different between the MG and BPPV w/o MG. The terms that were usually used by the patients to describe their vestibular symptoms during BPPV were evaluated under 13 headings [29]. As shown in Table 4, compared with the BPPV w/o MG, the MG reported significantly more frequent sensations of rocking back and forth (37.5% vs. 17.1%, *p* = 0.017); unsteadiness (71.8% vs. 39.0%, *p* < 0.001); feeling of fogginess in the head (40.6% vs. 20.3%, *p* = 0.021); and feeling like being drunk (50% vs. 29.6%, *p* = 0.030).

**Table 3:** According to ICVD, description of vertigo or dizziness by patients with BPPV

	**MG (N, %) (*N* = 64)**	**BPPV w/o MG (N, %) (*N* = 64)**	***p***
**Spontaneous vertigo**	29 (45.3)	27 (42.1)	0.858
**Visual stimulus-triggered vertigo**	31 (48.4)	17 (26.5)	0.017*
**Positional vertigo**	49 (76.5)	49 (76.5)	1,0
**Vertigo triggered by head movement**	49 (76.5)	53 (82.2)	0.509
**Dizziness triggered by head movement**	53 (82.8)	42 (65.6)	0.043*

*N* Number of participants, *ICVD* International Classification of Vestibular Disorders, *BPPV* Benign paroxysmal positional vertigo, *MG* Migraine group, *BPPV w/o MG* Benign paroxysmal positional vertigo without migraine group

^*^p < 0.05

**Table 4:** Various terms used by the participants to describe vertigo or dizziness during BPPV

	**MG (N, %) (*N* = 64)**	**BPPV w/o MG (N, %) (*N* = 64)**	***P***
Feeling of spinning around oneself	22 (34.3)	21 (32.8)	1.0
**Sensation of rocking back and forth**	24 (37.5)	11 (17.1)	0.017*
Feeling of the body leaning to the side	22 (34.3)	12 (18.7)	0.071
Sensation of swaying to both sides	21 (32.8)	15 (23.4)	0.325
**Unsteadiness**	46 (71.8)	25 (39.0)	< 0.001*
Feeling of emptiness in the head	24 (37.5)	19 (29.6)	0.454
Feeling of fogginess in the head	26 (40.6)	13 (20.3)	0.021
Impaired sense of place and time	8 (12.5)	6 (9.3)	0.777
**Feeling like being drunk**	32 (50)	19 (29.6)	0.030*
Feeling like car or sea sickness	21 (32.8)	15 (23.4)	0.325
Feeling like swimming in water	6 (9.3)	2 (3.1)	0.273
Feeling like walking in the air	18 (28.1)	11 (17.1)	0.205
Feeling like getting off a carousel	25 (39.0)	15 (23.4)	0.086

*N* Number of participants, *BPPV* Benign paroxysmal positional vertigo, *MG* Migraine group, *BPPV w/o MG* Benign paroxysmal positional vertigo without migraine group

^*^p < 0.05

### Motion sickness

The prevalence of motion sickness was significantly higher in the MG than in the BPPV w/o MG (82.9% vs. 54.7%, *p* < 0.001) .

### Comparison of clinical scales

#### Comparison of the MG with the BPPV w/o MG

Table 5 displays the baseline and 1–month follow-up VSS, VDI-SS, VDI-HRQoLS, BAI, and BDI scores for both groups. The median VSS score was significantly higher in the MG than in the BPPV w/o MG at baseline [16.5 (12; 27) vs. 9 (5.75; 15), *p* < 0.001] and on one-month follow-up [8 (5; 14.25) vs. 2 (0; 3), *p* < 0.001]. However, the change in VSS the scores from baseline to one-month follow-up was not significantly different between the groups [7.5 (4; 13) vs. 7 (4; 11.25), *p* = 0.939].

**Table 5:** Clinical scales in the MG and BPPV w/o MG groups at baseline and on the first month of follow-up

	**MG (*n* = 64)**	**BPPV w/o MG (*n* = 64)**	***P***
**VSS**			
**Baseline Median (IQR)**	16.5 (12; 27)	9 (5.75; 15)	< 0.001*
**Mild impairment (n, %)**	44 (68.7)	60 (93.7)	< 0.001*
**Severe impairment (n, %)**	20 (31.2)	4 (6.2)	
**One-month follow-up Median (IQR)**	8 (5; 14.25)	2 (0; 3)	< 0.001*
**Mild impairment (n, %)**	56 (87.5)	64 (100)	0.010*
**Severe impairment (n, %)**	8 (12.5)	0	
**Change Median (IQR)**	7.5 (4; 13)	7 (4; 11.25)	0.939
**VDI-SS**			
**Baseline Median (IQR)**	61.5 (54.25; 73)	80.5 (69; 87.25)	< 0.001*
**One-month follow-up Median (IQR)**	83 (72.75; 89)	95 (91.5; 98)	< 0.001*
**Change Median (IQR)**	-17 (-25.5; -7.75)	-13.5 (-24; -7)	0.507
**VDI-HRQoLS**			
**Baseline Median (IQR)**	81 (69; 92)	95 (87.5; 99)	< 0.001*
**One-month follow-up Median (IQR)**	92 (79.5; 97.25)	99.5 (97; 100)	< 0.001*
**Change Median (IQR)**	-5 (-14; -1.75)	-3 (-7.5; 0)	0.122
**BAI**			
**Baseline Median (IQR)**	12 (6.5; 19.25)	4.5 (1.75; 14)	< 0.001*
**Normal (n, %)**	20 (31.2)	40 (62.5)	0.003*
**Mild (n, %)**	21 (32.8)	10 (15.6)	
**Moderate (n, %)**	12 (18.7)	10 (15.6)	
**Severe (n, %)**	11 (17.1)	4 (6.2)	
**One-month follow-up Median (IQR)**	8 (3; 16)	2 (0; 6)	< 0.001*
**Normal (n, %)**	19 (31.1)	40 (62.5)	0.001*
**Mild (n, %)**	18 (29.5)	10 (15.6)	
**Moderate (n, %)**	19 (31.1)	14 (21.8)	
**Severe (n, %)**	5 (8.1)	0	
**Change Median (IQR)**	3 (1; 6)	2.5 (1; 5)	0.755
**BDI**			
**Baseline Median (IQR)**	8 (5; 14)	2 (0; 8.25)	< 0.001*
**Normal (n, %)**	36 (56.2)	49 (76.5)	0.085
**Mild (n, %)**	16 (25)	10 (15.6)	
**Moderate (n, %)**	11 (17.1)	5 (7.8)	
**Severe (n, %)**	1 (1.5)	0	
**One-month follow-up Median (IQR)**	7 (2; 11.25)	1 (0; 5)	< 0.001*
**Normal (n, %)**	43 (67.1)	56 (87.5)	0.038*
**Mild (n, %)**	16 (25)	6 (9.3)	
**Moderate (n, %)**	11 (17.1)	2 (3.1)	
**Severe (n, %)**	2 (3.1)	0	
**Change Median (IQR)**	0.5 (0; 3)	0 (0; 2)	0.122

*N* Number of participants, *MG* Migraine group, *BPPV w/o MG* Benign paroxysmal positional vertigo without migraine group, *VSS* Vertigo symptom scale, *VDI-SS* Vertigo dizziness imbalance symptom scale, *VDI-HRQoLS* Vertigo dizziness imbalance health-related quality of life scale, *BAI* Beck anxiety inventory, *BDI* Beck depression inventory, *IQR* Interquartile range as Q1:Q3

^*^p < 0.05

Impairment was classified as mild or severe based on the median VSS scores. Severe impairment (i.e., higher VSS scores) was more prevalent in the MG than in the BPPV w/o MG both at baseline (*p* < 0.001**)** and on one- month follow-up (*p* = 0.010**)**. At baseline, the percentage of patients with mild scores was significantly higher in the BPPV w/o MG than in the MG [68.7% (*n* = 44) vs. 93.7% (*n* = 60)], whereas that of patients with severe scores was significantly higher in the MG than in the BPPV w/o MG [31.2% (*n* = 20) vs. 6.2% (n = 4), *p* < 0.001]. Notably, one-third of the MG had severe VSS scores at baseline. By the first month of follow-up, severe VSS scores were not observed in the BPPV w/o MG but were persistent in 12.5% (*n* = 8) of the MG (*p* = 0.010).

The median VDI-SS score was significantly lower in the MG than in the BPPV w/o MG at baseline [61.5 (54.25; 73) vs. 80.5 (69; 87.25), *p* < 0.001] and on one-month follow-up [83 (72.75; 89) vs. 95 (91.5; 98), *p* < 0.001]. However, the change in VDI-SS scores from baseline to one-month follow-up did not show a significant difference between the groups [-17 (-25.5; -7.75) vs. -13.5 (-24; -7), *p* = 0.507].

The median VDI-HRQoLS scores were significantly lower in the MG than in the BPPV w/o MG at baseline [81 (69; 92) vs. 95 (87.5; 99), *p* < 0.001] and on one-month follow-up [92 (79.5; 97.25) vs. 99.5 (97; 100), *p* < 0.001]. However, there was no significant difference between the groups in terms of the change in the VDI-HRQoLS scores from baseline to the first month of follow-up [-5 (-14; -1.75) vs. -3 (-7.5; 0), *p* = 0.122].

The median BAI scores were significantly higher in the MG than in the BPPV w/o MG at baseline [12 (6.5; 19.25) vs. 4.5 (1.75; 14), *p* < 0.001] and on one-month follow-up [8 (3; 16) vs. 2 (0; 6), *p* < 0.001]. However, there was no significant difference between the groups in terms of the change in the BAI scores from baseline to the first month of follow-up [3 (1; 6) vs. 2.5 (1; 5), *p* = 0.755].

Using a categorical distribution (i.e., normal, mild, moderate, or severe), the BAI scores were significantly different between two groups at baseline (*p* < 0.001) and on one-month follow-up (*p* < 0.001). At baseline, the BAI score was normal in 62.5% (*n* = 40) of the BPPV w/o MG and in 31.2% (*n* = 20) of the MG showed. On follow-up after one month, the distributions of patients with normal and mild BAI scores were similar to those at baseline, but the subgroups of patients with moderate and severe BAI scores were higher in the MG (39.2%, *n* = 24) than in the BPPV w/o MG (21.8%, *n* = 14) (Table 5).

The median BDI scores were significantly higher in the MG than in the BPPV w/o MG at baseline [8 (5; 14) vs. 2 (0; 8.25), *p* < 0.001] and on the one-month follow-up [7 (2; 11.25) vs. 1 (0; 5), *p* < 0.001]. However, the change in the BDI scores from baseline to the first month of follow-up was not significantly different between the two groups [0.5 (0; 3) vs. 0 (0; 2), *p* = 0.122]. Using a categorical distribution, the BDI scores did not differ between the two groups at baseline (*p* = 0.084); however, on the first month, the number of patients who had normal scores was lower in the MG than in the BPPV w/o MG (67.1% vs. 87.5%, *p* = 0.038) (Table 5).

### Comparison of the vestibular migraine and nonvestibular migraine groups

Table 6 demonstrates the comparison of the clinical scales between the VM and nonVM groups. The median VSS was not significantly different between the two groups at baseline [19.5 (12; 27.75) vs. 15 (11.25; 24.25), *p* = 0.385]. However, on the first month, the median VSS score was significantly higher in the VM group than in the nonVM group [12 (6.5; 17) vs. 6 (4.25; 10.75), *p* = 0.043]. The change in the VSS scores between the two time points was not significantly different between the two groups (*p* = 0.411). Furthermore, the subgroupings to mild and severe impairment were not different between the two groups in the two time points.

**Table 6:** Clinical scales in the VM and Non-VM groups at baseline and on the first month of follow-up

	**VM Group** (*N* = 26)	**Non-VM Group** (*N* = 38)	***P***
**VSS**			
**Baseline Median (IQR)**	19.5 (12; 27.75)	15 (11.25; 24.25)	0.385
**Mild impairment (n, %)**	16 (61.5)	28 (73.6)	0.450
**Severe impairment (n, %)**	10 (38.4)	10 (26.3)	
**One-month follow-up Median (IQR)**	12 (6.5; 17)	6 (4.25; 10.75)	0.043*
**Mild impairment (n, %)**	21 (80.7)	35 (92.1)	0.336
**Severe impairment (n, %)**	5 (19.3)	3 (7.9)	
**Change Median (IQR)**	6.5 (2; 13)	8 (4.25; 12)	0.411
**VDI-SS**			
**Baseline Mean (SD)**	61.3 (15.0)	62.3 (16.0)	0.793
**One-month follow-up Median (IQR)**	79 (64.25; 86.5)	85.5 (77.5; 89.75)	0.074
**Change Mean (SD)**	- 14.3 (14.3)	- 18.7 (16.5)	0.262
**VDI-HRQoLS**			
**Baseline Median (IQR)**	73 (61; 85.75)	89 (71.5; 94.75)	0.003*
**One-month follow-up Median (IQR)**	85 (73; 92.75)	94 (87; 98)	0.005*
**Change Median (IQR)**	-7 (-14; -2.5)	-3 (-9; -1.25)	0.230
**BAI**			
**Baseline Median (IQR)**	15 (8.25; 22.75)	11 (4.25; 18.75)	0.233
**Normal (n, %)**	6 (23.0)	14 (36.8)	0.677
**Mild (n, %)**	9 (34.6)	12 (31.5)	
**Moderate (n, %)**	6 (23.0)	6 (15.7)	
**Severe (n, %)**	5 (19.2)	6 (15.7)	
**One-month follow-up Median (IQR)**	10 (5.25; 18.25)	6 (2; 13.5)	0.089
**Normal (n, %)**	5 (19.2)	14 (36.8)	0.424
**Mild (n, %)**	10 (38.5)	9 (23.6)	
**Moderate (n, %)**	9 (34.9)	12 (31.6)	
**Severe (n, %)**	2 (7.7)	3 (7.9)	
**Change Median (IQR)**	3 (1.25; 5.75)	2 (1; 6.75)	0.858
**BDI**			
**Baseline Median (IQR)**	9.5 (7; 13.5)	7 (3.25; 13.75)	0.258
**Normal (n, %)**	13 (50.0)	23 (60.5)	0.641
**Mild (n, %)**	8 (30.8)	8 (21.0)	
**Moderate (n, %)**	5 (19.0)	6 (15.8)	
**Severe (n, %)**	0	1 (2.6)	
**One-month follow-up Median (IQR)**	8.5 (7; 11.75)	4.5 (1; 9.75)	0.029*
**Normal (n, %)**	15 (57.7)	28 (73.7)	0.554
**Mild (n, %)**	8 (30.8)	8 (21.0)	
**Moderate (n, %)**	2 (7.7)	1 (2.6)	
**Severe (n, %)**	1 (3.8)	1 (2.6)	
**Change Median (IQR)**	0.5 (-1; 3)	0.5 (0; 4.75)	0.198

*N* Number of participants, *VM* Vestibular migraine, *Non-VM* Migraine without vestibular migraine, *VSS* Vertigo symptom scale, *VDI-SS* Vertigo dizziness imbalance symptom scale, *VDI-HRQoLS* Vertigo dizziness imbalance health-related quality of life scale, *IQR* Interquartile range as Q1:Q3

^*^p < 0.05

The VDI-SS scores were not significantly different between the VM and nonVM groups at baseline [61.3 (15.0) vs. 62.3 (16.0), *p* = 0.793] and on one-month follow-up [79 (64.25; 86.5) vs. 85.5 (77.5; 89.75), *p* = 0.074]. However, the median VDI-HRQoLS indicated a significantly higher impairment in the VM group than in the nonVM group both at baseline [73 (61; 85.75) vs. 89 (71.5; 94.75), *p* = 0.003] and on one-month follow-up [85 (73; 92.75) vs. 94 (87; 98), *p* = 0.005].

The mean/median scores and categorical distributions of the BAI did not show any significant differences at baseline and on one-month follow-up between the two groups. Moreover, the median BDI scores were not significantly different between the VM and nonVM groups at baseline [9.5 (7; 13.5) vs. 7 (3.25; 13.75), respectively, *p* = 0.233]. In one-month follow-up, the BDI score did not show any statistical difference between the VM group than in the nonVM group [8.5 (7; 11.75) vs. 4.5 (1; 9.75), *p* = 0.089]. In addition to that, the categorical distribution of the BDI in the groups was not significantly different between the two groups.

### Correlation analysis for vestibular migraine

In the VM group, the HIT-6 score had a moderate positive correlation with the VSS (*p* = 0.018, rho = 0.457) and a moderate negative correlation with the VDI-SS (*p* = 0.010, rho =  − 0.491). Moreover, MIDAS was strongly correlated with the VSS (*p* = 0.001, rho = 0.60) and moderately correlated with the VDI-SS (*p* = 0.002, rho =  − 0.56).

In the nonVM group, the HIT-6 score had a weak relationship with the BAI (*p* = 0.024, rho = 0.36) and a moderate relationship with the BDI (*p* = 0.006, rho = 0.431).

## Dıscussıon

In this study, during at both the diagnosis and on the one-month follow-up for BPPV, patients with migraine experienced more severe vestibular symptoms and a greater adverse impact on their quality of life compared to those without migraine. At all assessment points, the MG who exceeded the VSS cut-off score associated with severe vestibular symptoms was statistically more prevalent to those without migraine. The similar rate of improvement in both groups indicated that suffering from migraine did not negatively affect the recovery from BPPV within a one-month period. Patients diagnosed with VM exhibited a frequent family history with migraine and longer migraine duration, along with higher baseline MIDAS scores and increased MHDs at the one-month follow-up, when compared to patients without VM. Individuals with VM exhibited higher VVS scores during the follow-up period and experienced significantly more impaired health-related quality of life at all assessment points compared to those without VM. Finally, in patients with VM, the MIDAS demonstrated a strong correlation with the VSS, and a significant negative correlation with the VDI-SS.

Studies have shown that BPPV was more common in individuals with migraine than in healthy controls [14, 30, 31] and that having migraine increased the risk of BPPV [13]. Migraine was relatively frequent in women and young patients with a history of BPPV [32]. Ishiyama et al. [31] demonstrated that in patients with BPPV without migraine, the age of onset age was older, reaching a peak in the eighth decade. Nearly half (47%) of the patients who experienced BPPV before the age of 50 years had a history of migraine. Faralli et al. [33] showed that the mean age of BPPV onset was 39 ± 9.2 years in patients with migraine and 53 ± 7.3 years in individuals without migraine. In alignment with prior research, we observed that the MG was significantly younger, compared with the BPPV w/o MG.

In a comprehensive retrospective study in the United States, the factors that affected the coexistence of migraine and BPPV were investigated using a substantial participant pool (*n* = 1481) [32]. The results showed that the self-reported prevalence of migraine among patients with BPPV was 25.8% (*n* = 382). The authors identified female sex, young age, history of previous BPPV, and absence of diabetes mellitus (DM) as the common comorbidities of migraine and BPPV. That study revealed a greater prevalence of coexistent BPPV and migraine in individuals who had a prior history of BPPV than in those who had no BPPV (OR 1.6, 95% CI 1.2–2.1, *p* < 0.002). Similarly, in our cohort, the patients were younger and were predominantly women in the MG than in the BPPV w/o MG. In other previous studies, vascular comorbidities, such as hypertension, DM, and hyperlipidemia, may have negative effects on the occurrence [34] or recurrence [35] of BPPV. In our study, hypertension was significantly more prevalent in the BPPV w/o MG than in the MG (*p* = 0.038); this may have been associated with relatively old age of the patients in the BPPV w/o MG. However, considering the limited number of participants, this finding should be interpreted with caution. Consistent with the literature [36], our study reported a significantly higher prevalence of a recent history of head trauma in the MG than in the BPPV w/o MG.

Most cases of BPPV develop in the posterior and horizontal canals. In our cohort, involvement of the PC was observed in 81.2% of the MG and 67.1% of the BPPV w/o MG, consistent with the literature [37]. The rate of BPPV recurrence has been reported to range from 7 to 56% [34, 38, 39]. In a study on general population, Luryi et al. [40] found a BPPV recurrence rate of 37%, an increased risk of recurrence in females and individuals who had a history of BPPV, and a previous history of BPPV as the most significant factor associated with BPPV recurrence [40]. In our study, most participants had a history of BPPV, although we did not find an increased rate of BPPV recurrence in the MG. The high rates of BPPV recurrence in this study might be related to the setting of a tertiary referral center. Similarly, Ishiyama et al. [31] reported high BPPV recurrence rates in both migraineurs (77%, *n* = 62) and nonmigraineurs (66%, *n* = 154).

In this present study, the prevalence of motion sickness was significantly higher in the MG than in the BPPV w/o MG and only tended to be higher in the VM group than in the nonVM group. In our previous study, more frequent headaches and more intense vestibular symptoms during caloric testing were observed in patients with migraine, particularly those who had motion sickness alone and those who had both migraine and motion sickness, than in patients without migraine and motion sickness [41].

In this study, the incidence of visually-induced vertigo and dizziness triggered by head movement was higher in the patients with migraine than in those without migraine. After a BPPV episode, patients may experience vertigo or dizziness in different forms and rates. In patients with VM, vestibular findings were also experienced in different ways [42]. Activation of the noradrenergic locus coeruleus and serotonergic dorsal raphe nucleus has been demonstrated in migraineurs [43]. These regions are important anatomical areas for modulating the intensity of sensory stimuli, such as light and sound [44]. We believed that the significantly higher incidence of visually-induced vertigo in migraineurs may be related to the modulation of the vestibuloocular reflex and spatial processing in the high cortical centers, which are thought to be affected in the pathophysiology of VM with BPPV.

In our study, the sensations of forward and backward swaying, unsteadiness, head heaviness, and a feeling of being drunk were significantly more frequent in migraineurs than in nonmigraineurs. These differences in the perception of vestibular symptoms highlighted the importance of taking the time to dig deep into the history to differentiate and diagnose vertigo [29].

Consistent with our hypothesis, we observed that the assessment scales for the severity of the vertigo symptoms and the feeling of dizziness were higher in migraineurs than in nonmigraineurs both at baseline and on the first month. In addition, we concluded that having migraine with BPPV had a greater impact on QoL at the time of BPPV diagnosis and on the first month of follow-up. In a cross-sectional study on patients with MWA, MWoA, CM, and healthy control subjects (*n* = 60 for each), the dizziness handicap inventory scores were significantly higher in the patients with migraine than in the healthy controls (*p* < 0.001) [45], and the disability was more pronounced in the patients with MWA and CM than in the patients with MWoA. However, we were unable to make a direct comparison between the results of that previous study and our study, because majority of our cohort comprised patients with EM, most of our patients had MWoA, and the number of patients with CM was limited.

In a recent study on 58 patients diagnosed as BPPV, the duration of dizziness following BPPV episodes was reported to be longer in patients with migraine than in patients without migraine [46]. In our study, the assessment scales for the severity of the vertigo symptoms and the feeling of dizziness were higher in migraineurs than in nonmigraineurs at baseline and after one month, but the MG and BPPV w/o MG showed similar improvements in the VSS, VDI-SS, and VDI-HRQoLS scores after one month. If both BPPV and migraine are thought to be risk factors for the development of PPPD, one of the shortcomings of this study is that the vertigo and dizziness that persist in migraine patients in the first month after BPPV were not re-evaluated at the end of the third month.

Both the BAI and BDI scores were higher in the MG than in the BPPV w/o MG at the baseline and on one-month follow-up. Psychiatric comorbidities reduce the QoL of patients with migraine and may complicate migraine management [47]. The findings of our study highlighted the importance of considering psychiatric comorbidities in migraineurs who are being diagnosed as BPPV and during the recovery process following BPPV.

In a recent prospective study that compared patients with VM (*n* = 50) and patients with migraine only (*n* = 35), the MIDAS, Visual Analogue Score, and BDI score were significantly higher in the latter (*p* < 0.05), whereas the Balance Confidence scores were significantly lower in the former (*p* < 0.001) [48]. These findings indicated that headache was more prominent in patients with migraine only, but vestibular complaints were more prominent in patients with VM. In our study, although many patients reported the frequency of EM, the MIDAS scores indicated a mild level of disability in all patients with migraine. Moreover, the MIDAS was significantly higher in the VM group than in the nonVM group. Notably, there was no increase in the number of MHDs in the MG after one month. This finding suggested that VM might influence migraine-related disability independently of headache frequency. Based on our correlation analysis in the VM group, the severity of vertigo and dizziness seemed to be related with the impact of headache and migraine-related disability. However, in the nonVM group, the impact of headache correlated with psychiatric comorbidities. Our findings highlighted the need to ask about headache and psychiatric comorbid conditions when managing patients with BPPV.

Our study had strengths and weaknesses. Although all interviews were conducted by the same neurology resident, some patients refused to participate and some were lost to follow-up, probably because the total duration of the baseline and follow-up interviews was 1.5 h. One of the strengths of our study was its prospective design that evaluated the severity of vestibular symptoms, QoL, and psychiatric comorbidities in patients with and without migraine using a comprehensive methodology. Additionally, the study assessed the course of BPPV and its effects on migraine frequency through one-month follow-up. Another strength was the subgroup analysis of patients with VM. However, it should be noted that VM can be confused with BPPV, both clinically and in terms of the VNG findings. During acute VM attacks, spontaneous and positional nystagmus alone or in combination can be observed [49]. In the study by Beh et al. [50], the incidence of positional vertigo was as high as 25.2% during VM attacks but was only13% outside of the attacks. In our study, the patients were evaluated for VM by both neurology and ENT specialists. VM was diagnosed based on clinical interview, whereas BPPV diagnosis was confirmed by VNG. PPPD patients were not included in the study. All patients were evaluated and VNG was performed by the same experts, and the clinical interviews were conducted by only one neurology resident; these supported the reliability of our findings.

Some limitations of our study need to be addressed. There were more women and younger participants in the MG than in the BPPV w/o MG. We did not have sex–age matched patients with BBPV without migraine. These inequalities might have affected our results. However, previous studies showed that compared with patients with BPPV alone, patients with BPPV with migraine were younger and had a preponderance of women. Our cohort reflected a real life setting in that way. Because our study was conducted at a tertiary referral center, it should be emphasized that our results may not reflect the findings in the general population, and caution should be exercised when generalizing our findings. Another weakness was the limited number of participants. Although the minimum recommended number of participants was achieved after a power analysis during the planning phase, the results may vary if the number of subjects is increased. Furthermore, majority of the participants had EM. The relationship between migraine and BPPV could have been more effectively evaluated if the participants were evenly divided into the EM and CM groups. In this study, 21.8% of our patients with migraine were under prophylactic treatment, which might have influenced the severity and course of BPPV symptoms in these patients. In our study, during the BPPV diagnosis, the number of MHDs in the preceding month was based on memory recall of the patients in the MG. Because these data did not involve objective tracking, the actual situation might not have been fully reflected. Moreover, our patients were not evaluated as two subgroups (i.e., canalolithiasis and cupulolithiasis). This omission may have affected the assessment of the severity of symptoms during BPPV and the disease course and prognosis, thereby, possibly affecting our results. In addition, a substantial proportion of patients in the MG exhibited VM (40.6%). Hence, it is plausible that the heightened vestibular morbidity observed in the BPPV group with migraine might be attributed to the supplementary vestibular symptoms present in the VM subgroup. Finally, PPPD is one of the most common reasons of chronic dizziness. For a diagnosis of PPPD, persistent symptoms need to be lasted for 3 months or longer. The cross-sectional nature of our study does not allow us to assess whether our patients developed PPPD. This underlines the need to be careful in interpreting our results and to keep PPPD in mind.

## Conclusions

Our findings highlighted the importance of asking patients with BPPV about migraine and psychiatric comorbidities. Prospective studies with a larger participant pool and a focused examination of how being migraine affects the onset, progression, and recovery of BPPV will offer valuable insights for physicians managing these prevalent conditions in the future.

### Supplementary Information

**Supplementary Material 1. **

## Data Availability

The datasets of the current study are available from the corresponding author upon reasonable request.

## Abbreviations

BPPV: Benign paroxysmal positional vertigo
MG: Migraine group
BPPV w/o MG: BPPV without migraine group
VSS: Vertigo Symptom Scale
VDI-SS: Vertigo Dizziness Imbalance Symptom Scale
VDI-HRQoLS: VDI Health-Related Quality of Life Scale
BAI: Beck Anxiety Inventory
BDI: Beck Depression Inventory
QoL: Quality of life
VM: Vestibular migraine
ENT: Ear nose throat
VNG: Videonistagmography
HIT-6: Headache Impact Test
MIDAS: Migraine Disability Assessment Scale,
MWA: Migraine with aura
MWoA: Migraine without aura
EM: Episodic migraine
CM: Chronic migraine
MHDs: Monthly headache days
DM: Diabetes mellitus
PPPD: Persistent postural perceptual dizziness
