# Supplementary material for: Assessing the impact of migraine on benign paroxysmal positional vertigo symptoms and recovery
Source: BMC Neurol. 2024 May 2;24:148. doi: 10.1186/s12883-024-03606-2 (PMC11064330; doi:10.1186/s12883-024-03606-2)
Supplement: Supplementary file 1 — Supplementary Material 1. [file 12883_2024_3606_MOESM1_ESM.docx]

**Appendix 1: Socio-Demographic and Clinical Characteristics Data Form**

**Sociodemographic Characteristics**

Name:

Protocol:

Age:

Gender: a) Female b) Male

Years of Education: … years (years of schooling)

Employment Status: a) Employed b) Unemployed c) Retired d) Student.

What are the comorbid diseases of the patient?

What are the medications the patient is currently taking regularly?

Does the patient smoke?

Does the patient consume alcohol?

**Benign Paroxysmal Positional Vertigo (BPPV)**

Which ear is affected in BPPV? Right/Left/Both

Which canal is affected in BPPV? Posterior/ Anterior/ Horizontal/ Mixed

Does the patient have a history of previous BPPV episode/s? a) Yes b) No

Risk Factors: Trauma, infection, migraine, motion sickness, prolonged rest, exercise, heavy alcohol consumption, vitamin D deficiency

How many maneuvers were performed before repositioning occurred in the patient's BPPV episode?

Does the patient currently have symptoms consistent with motion sickness? a) Yes b) No

Does the patient have a history consistent with motion sickness in her/his childhood? a) Yes b) No

**Migraine**

Has the patient been previously diagnosed with migraine? a) Yes b) No

What is the duration of migraine? (months/years)

Is there a history of migraine in the patient’s family? a) Yes b) No

What is the frequency of headache days in the past month for the patient?

Is the patient currently on migraine prophylactic treatment?

How long has the patient been using prophylactic treatment for migraine?

How long has the patient been using prophylactic treatment for migraine? a) Yes b) No

If so, which classes of medication has the patient used for migraine preventive treatment?

How many analgesics are taken by the patient at the last month?

How many triptans are taken by the patient at the last month?

Does the patient meet the diagnostic criteria for vestibular migraine? a) Yes b) No

**Vestibular Symptoms Defined by International Classification of Vestibular Disorders**

Spontaneous vertigo as a false motion sensation of self or surrounding Visually induced vertigo
Positional vertigo (after a change of head position)
Head motion-induced vertigo (during head motion)
Head motion-induced dizziness (sensation of disturbed spatial orientation)

**When describing vertigo, ask patients about:**

Sensation of spinning around oneself

Swaying forward and backward

Tilting of the body to the side

Swaying from side to side

Unsteadiness

Sensation of emptiness in the head

Fuzziness in the head

Distorted sense of place and time

Feeling intoxicated

Feeling motion sickness in a car or on the sea

Feeling like swimming in water

Walking in the air

Getting off a rotating amusement ride
